# Mind-Wandering Tends to Occur under Low Perceptual Demands during Driving

**DOI:** 10.1038/srep21353

**Published:** 2016-02-17

**Authors:** Chin-Teng Lin, Chun-Hsiang Chuang, Scott Kerick, Tim Mullen, Tzyy-Ping Jung, Li-Wei Ko, Shi-An Chen, Jung-Tai King, Kaleb McDowell

**Affiliations:** 1 Brain Research Center, National Chiao Tung University, MIRC 416, 1001 University Road, Hsinchu 30010, Taiwan; 2 University of Technology Sydney, 15 Broadway, Ultimo, New South Wales 2007, Australia; 3 Translational Neuroscience Branch, U.S. Army Research Laboratory, 459 Mulberry Point Road, Aberdeen Proving Ground, MD 21005-5425, USA; 4 Swartz Center for Computational Neuroscience, University of California, San Diego, 9500 Gilman Drive, La Jolla, CA 92093-0559, USA; 5 Department of Biological Science and Technology, National Chiao Tung University, Hsinchu, Taiwan

## Abstract

Fluctuations in attention behind the wheel poses a significant risk for driver safety. During transient periods of inattention, drivers may shift their attention towards internally-directed thoughts or feelings at the expense of staying focused on the road. This study examined whether increasing task difficulty by manipulating involved sensory modalities as the driver detected the lane-departure in a simulated driving task would promote a shift of brain activity between different modes of processing, reflected by brain network dynamics on electroencephalographic sources. Results showed that depriving the driver of salient sensory information imposes a relatively more perceptually-demanding task, leading to a stronger activation in the task-positive network. When the vehicle motion feedback is available, the drivers may rely on vehicle motion to perceive the perturbations, which frees attentional capacity and tends to activate the default mode network. Such brain network dynamics could have major implications for understanding fluctuations in driver attention and designing advance driver assistance systems.

Safe driving requires that drivers sustain attention and alertness behind the wheel. Policy-makers and researchers have focused on reducing external driver distraction (e.g., laws requiring hands-free use of cell phones) as well as on minimizing the probability that a driver will fall asleep at the wheel. However, even when a driver is alert and in the absence of external distractions fluctuations in attention are inevitable^1^. Such fluctuations, often referred to as mind-wandering^2^,^3^, impair drivers’ ability to maintain consistent speed and lane integrity and respond to emergency situations^4^,^5^ and is likely a contributing factor in more than half of all car crashes^6^. Hence, a comprehensive understanding of brain networks underlying changes in alertness may lead to new insights for augmenting human performance and lowering the risk of accidents.

Given that humans have a limited global workspace or attentional capacity^7^,^8^, the degree to which we prioritize, weigh, or bias our attention toward the immediate internal or external environment varies depending on current goals, motivation, levels of alertness, the complexity of the task and the context under which it is being performed. Thus, the brain’s large-scale networks may interrelate in a dynamic, flexible and adaptive manner to function either cooperatively or competitively depending on circumstances or context^8^,^9^,^10^. The default mode network^11^ is predominantly activated and functionally coherent when one is in a resting state and no explicit task is being performed, when attention is directed toward internal states, thought processes, memories, or feelings, and during performance of a highly over-learned or low-demand task (e.g., driving down a highway under low traffic conditions in a familiar or monotonous environment). Conversely, the task-positive^12^ or frontal-parietal attention network^13^,^14^ is predominantly activated and functionally coherent during performance of tasks requiring attention to sensory-perceptual information from the external environment, especially during performance of a novel or high-demand task (e.g., driving under high traffic conditions in an unfamiliar environment). Therefore, we posit that changes in driving task demand may promote a shift of brain activity between these two modes of processing. Furthermore, fluctuations in performance during driving may be related to fluctuations in internal and external attentional states associated with dynamic changes in functional coupling between the brain’s default and task-positive networks.

To test this prediction, we altered perceptual demands by manipulating sensory inputs provided to participants in an event-related lane-departure paradigm^15^ in a driving simulator using a six degree-of-freedom vehicle motion simulator (see Fig. 1a,b and Supplementary Information for more details). In one condition (K^+^) both visual and vehicle motion feedbacks were provided to drivers and in a comparative condition (K^−^) only visual feedback was provided. This is because vehicle motion provides kinesthetic and vestibular sensory inputs, which are the most relevant sources of sensory input used by humans for control purposes during driving following visual input^16^. Previously, we^17^ have shown that simulated driving in the absence of vehicle motion feedback imposes a relatively more perceptually-demanding task than driving with such feedback, as evidenced by event-related theta activity (an indicator of cognitive demand^18^).

In the present study, we recorded electroencephalographic (EEG) signals while ten participants performed the sustained-attention driving task for 60 min. Participants were required to maintain their attention, detect any lane-departure, and promptly steer the vehicle back to the center of the cruising lane. The reaction time (RT) to the perturbation was determined as a measure of the subjects’ task performance in the simulated task. Using a repeated-measures design, the participants completed two driving sessions on different days in a counterbalanced order (see Methods).

We explored the differences in brain network dynamics during the pre-stimulus period (Fig. 1c) between the K^+^ and K^−^ sessions by applying independent component analysis^19^ and Granger causality analysis^20^ to model directed information transfer, or *effective connectivity*^21^ (see Methods), between sources of EEG activity (see Supplementary Fig. 5) localized to the anterior cingulate cortex (ACC), midcingulate cortex (MCC), posterior cingulate cortex (PCC), sensorimotor cortex (SMC), and extrastriate cortex (ESC) which were selected based on their central roles in the functional brain networks^22^,^23^.

## Results

**Figure 2:**
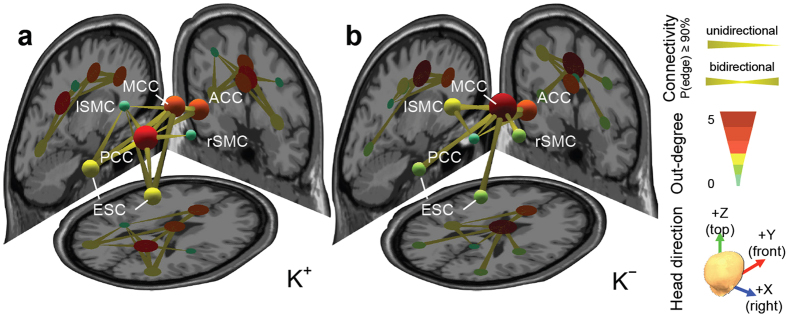
Brain network dynamics. Effective connectivity between EEG independent processes estimated under (**a**) K^+^ and (**b**) K^−^ conditions. Three-dimensional plot is formed with three anatomical MRI slices (left: sagittal view; right: coronal view; bottom: horizontal view) as background. Node represents anatomical location of each independent process, localized using a single, or dual-symmetric equivalent-current dipole model based on a four-shell spherical head model. Color of nodes represents degree of outflow. Edges represent causality directions. P(edge) ≥ 0.9 indicates that over 90% of participants have a significantly non-zero connectivity magnitude in that edge (see Supplementary Table 3). Out-degree indicates the number of outgoing edges.

Analysis of effective connectivity of the K^+^ session (Fig. 2a) revealed that a dominant causal hub was located in PCC, which received information from ACC and MCC, while sending information to MCC, bilateral SMC, and ESC. The minor hubs were located in ACC, with unidirectional information transfer to left SMC and PCC, and in MCC, with bidirectional information transfer to PCC and ESC. In the K^−^ session (Fig. 2b), the dominant causal hub shifted to MCC with bidirectional information exchange with all other brain regions. Compared to the result under the K^+^condition, the causal influence of MCC and PCC on other regions significantly increased (MCC → lSMC) and decreased (PCC → ACC and PCC → rSMC) respectively while the influence of ACC on remaining brain regions remained unchanged under the K^−^ condition (McNemar’s test, FDR-adjusted *p* < 0.05, see Supplementary Table 4). Additionally, under both K^+^ and K^−^ conditions, bilateral SMC and ESC contributed weakly to the causal network, exchanging information unidirectionally with PCC under the K^+^ condition (PCC → SMCs and PCC → ESC) and bidirectionally with MCC under the K^−^ condition (MCC ↔ SMCs and MCC ↔ ESC). Taken together, depriving participants of crucial information regarding the imminent lane departure promoted this alteration of pre-stimulus EEG activity.

**Figure 3:**
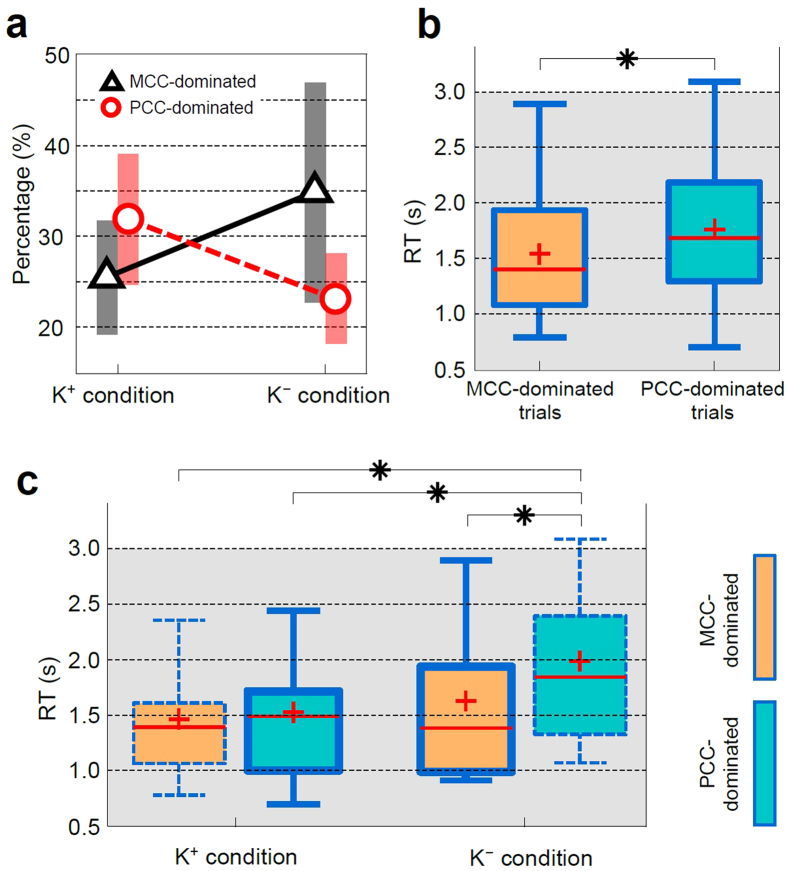
Differences of task performance between causal hubs. (**a**) Percentages of MCC- and PCC-dominated trials under K^+^ and K^−^ conditions. Each bar reflects 95% confidence intervals. (**b**) Box plots of the RT of the trials with MCC-dominated and PCC-dominated networks. Shown are median (horizontal line), mean (cross), 25% and 75% quartiles (box), and minimum and maximum values (error bars). (**c**) Box plots of the RT of the trials with MCC-driven and PCC-driven networks estimated in K^+^ and K^−^sessions. The bold boundary denotes the dominant network according to the connectivity shown in Fig. 2a,b. The comparison of the RT between networks and sessions were performed by Wilcoxon signed-rank test (FDR-corrected *p* < 0.05).

Brain networks are hypothesized to be affected to a varying extent depending on the current level of engagement in the driving task. To estimate the extent of the network change throughout the driving tasks, single-trial Granger causality analysis was performed on each pre-stimulus EEG trial of the K^+^ and K^−^ sessions (see Methods). All trials were sorted by the causal hub, i.e., the region with the highest number of causal outflow connections (i.e., out-degree). The results showed that, across conditions, there was a mixture of MCC-dominated and PCC-dominated trials (Fig. 3a). The mean percentages of MCC-dominated trials and PCC-dominated trials were 25.5% and 31.9%, respectively, under the K^+^ condition and 34.9% and 23.1%, respectively, under the K^−^ condition. A repeated measures ANOVA analysis further revealed that the interaction effect of sensory feedback and out-degree of brain networks was significant (*p* = 0.015).

Next, we collapsed across feedback conditions and sorted trials by PCC- and MCC-dominant network outflow and examined RTs associated with each network (see Methods). The results revealed that RTs were significantly longer for trials dominated by PCC outflow (Fig. 3b). The difference of RTs between networks reached 286 ms (see Supplementary Table 5). This evidence suggested that the brain causal activity driven by MCC is positively correlated with task performance. Conversely, the brain causal activity driven by PCC is negatively correlated with task performance. Then we examined RTs sorted by PCC- and MCC-dominated network outflow in each condition and compared RTs between feedback conditions. An interaction was observed such that RTs were longer for trials dominated by PCC in the K^−^ condition but did not differ in the K^+^ condition (Fig. 3c).

**Figure 4:**
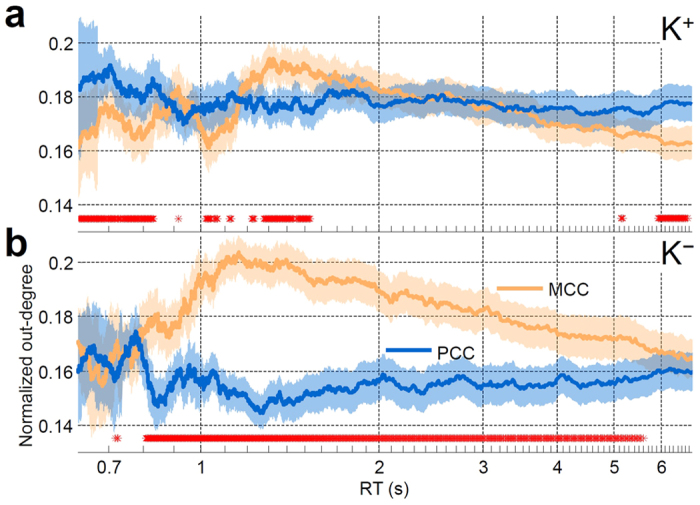
Change in out-degree across different task performance. RT-sorted outflow dynamics of MCC (yellow trace) and PCC (blue trace) under (**a**) K^+^ and (**b**) K^−^ conditions. For each trial, the out-degree was normalized by dividing the sum of significant outflow links of all brain regions. Then, the normalized out-degrees are horizontally stacked according to the RT from fast to slow and smoothed with a central moving average filter (window size: 10% of trials; window step: 1 trial)(see Methods). Red asterisks indicate that the difference between two traces is significant. Significant at FDR-adjusted *p* < 0.05.

Finally, we sorted trials by RTs within each feedback condition and examined changes in network dynamics (out-degree of PCC- and MCC-dominant networks) as a function of transient changes in performance (see Methods). The out-degrees of PCC were fairly stable across different RTs at 0.18 under K^+^ and 0.16 under K^−^ condition (Fig. 4), respectively. However, the out-degrees of MCC increased from fast RT to intermediate RT and then decreased from intermediate RT to slow RT under both K^+^ and K^−^ conditions. This inverted U-shaped change of MCC outflow specifically occurred in the connectivity of MCC → ACC under both K^+^ and K^−^ conditions, but occurred in the connectivity of MCC → bilateral SMC only under the K^−^ condition (see Supplementary Fig. 6). In most trials under the K^+^ condition, the out-degrees of PCC were higher than those of MCC when RT < 0.8 s, 1 s < RT < 1.1 s, and RT > 6 s (Fig. 4a), leading to the overall PCC-dominant network shown in Fig. 2a. Conversely, most trials of the K^−^ condition showed that the out-degrees of MCC were higher than those of PCC when 1 s < RT < 2 s (Fig. 4b), leading to the overall MCC-dominant network shown in Fig. 2b.

## Discussion

One hypothesis to account for these findings is the perceptual decoupling hypothesis^2^,^24^, which posits that attention to sensory information in the environment is down-weighted to enable internal thought processes to proceed without interference from external stimuli. Driving is essentially a complex continuous tracking task requiring bottom-up processes for the multisensory integration of information from the external environment as well as top-down modulatory influences based on the driver’s internal goals, strategies, and current intentions. Additionally, driving is a highly over-learned task for experienced drivers, such that they are able to drive with high levels of automaticity especially when driving under low demand conditions. Under such conditions, drivers are able to engage in internally-directed self-generated mental activity while simultaneously monitoring the external environment and maintaining control of the vehicle, all with relative ease.

The present results further showed that participants are more likely to decouple their attention to sensory information during the pre-stimulus time period in the environment under relatively lower task demand conditions, apparently even when sensory information is highly salient. The participants exhibited significantly more causal outflow from the MCC (node of task-positive network) versus PCC (node of default mode network) node during driving while deprived of salient sensory information (K^−^ condition in this study). Removing vehicle motion feedback during simulated driving deprives the driver of salient sensory information and, therefore, should impose relatively greater perceptual and executive demands on the driver to maintain vehicle control. Conversely, drivers succumbed to greater levels of inattention when the task is less demanding (K^+^ condition), as evidenced by more causal outflow from the PCC versus MCC node when vehicle motion feedback was provided.

In both K^+^ and K^−^ conditions, the out-degree of MCC has an inverted U-shaped trend (Fig. 4). Previous studies have verified that compensatory brain resources^25^ are required to maintain performance, particularly in a task with high cognitive demand. Increased causal outflow from MCC to bilateral SMC observed at the intermediate level of task performance (see Supplementary Fig. 6) corroborates these findings and suggests that the subjects might raise their attentional efforts to maintain vehicle control^26^ while fighting off waves of boredom or fatigue. MCC is dominant when the participants were relatively more engaged in the task and attempting to stay engaged in the task. Additionally, in the tail end of the distribution of RTs (RT ≥ 5 s), the subjects were nearly non-responsive, and MCC outflow markedly reduced below or comparable to the outflows of PCC in both K^+^ and K^−^ conditions, which also supports the perceptual decoupling hypothesis^2^,^24^. It is worth noting that the effects of vehicle motion feedback (K^+^ vs. K^−^) on the brain and behavior might not be limited to the allocation of attentional resources. The results of this study cannot rule out the possibility that the differences between K^+^ and K^−^ were associated with the involvement of different sensory modalities as well as the subjective sensation of movement.

Our previous studies^17^,^27^ have shown that EEG spectral changes are associated with task performance (see Supplementary Fig. 7). Theta power (4**–**7 Hz) increases as performance declines and the driver’s attention deteriorates. Consistent with these findings, in the present study the knee-point of the spectrum-vs.-RT curve occurred at 1.8 s where the spectral augmentation reached a plateau under K^+^ condition, matching the result of network analysis where the out-degrees of MCC reduced to the level comparable to those of PCC. On the other hand, the spectrum-vs-RT curve reached a plateau at 4 s, matching the result of network analysis where the out-degrees of MCC reduced to the level comparable to those of PCC at ~4 s. In the RT range of 1-4 s, the participants exhibited higher theta power in the MCC under the K^+^ versus the K^−^ condition. Association between lower out-degree and higher theta band power of MCC in the K^+^ condition suggests that alertness is relatively low when the vehicle motion feedback is available. Additionally, in the K^+^ condition, accompanying the continuously monotonic theta augmentation is the alpha suppression (see Supplementary Fig. 7c,d). This opposite trend of theta and alpha activities is a neural marker of low alertness during mind wandering^28^.

Based on the evidence provided in this study, neural oscillatory activities of decreased alertness become active when the perceptual demand decreases. A number of advanced driver assistance systems have created new opportunities in facilitating the driving task and enhancing road safety. Many of them utilize warning signals presented in different sensory modalities to alert drivers to potential hazards. There are valid concerns that over-reliance on in-vehicle technologies may result in reduced situational awareness and low task engagement, rather than enhancing road safety. Hence, potential hazards caused by reduced perceptual demand should be considered in developing advanced driver assistance systems in modern cars.

## Methods

Previously, we examined brain dynamics of participants during simulated driving with and without kinesthetic feedback using an event-related lane-departure paradigm^17^. Specifically, we derived brain sources in frontal, central, motor, parietal, and occipital cortical regions using independent component analysis^19^ and examined variations in component spectra between kinesthetic feedback conditions across different levels of performance (optimal, sub-optimal, and poor), as determined by the distribution of reaction times (RT) to random lane departure events. We observed a significant interaction, such that RTs in the optimal range of the distribution were faster in the kinesthetic feedback condition versus the no-kinesthetic feedback condition, but for the tail of the distribution of RTs (i.e., sub-optimal and poor ranges) RTs were slower in the kinesthetic feedback condition. In contrast to the no-kinesthetic feedback environment with visual input only, kinesthetic feedback reduced theta-power augmentation in the central and frontal components when preparing for action and error monitoring. The event-related theta activity typically is associated with the level of cognitive demand^18^,^29^ and the amount of attentional resources^30^ for planning the required motor action^31^ and integrating the sensorimotor function^32^, showing that the drivers succumbed to greater levels of inattention when kinesthetic feedback was provided, as evidenced by both RTs and brain network dynamics.

Overall, these findings suggest that kinesthetic feedback functioned to facilitate performance when the drivers were optimally alert (operationally defined as the fastest RTs), but when their alertness levels deteriorated (defined as intermediate and slow RTs) the kinesthetic feedback actually functioned to worsen performance. These findings might be correlated with the perceptual decoupling account of mind-wandering, which posits that participants are more likely to decouple their attention to sensory information in the environment under relatively lower task demand conditions, even when sensory information is highly salient.

Hence, in the present simulated driving study, we manipulated sensory inputs provided to drivers and investigated the differences of brain network dynamics, derived from EEG signals, between different sensory feedback conditions (K^+^, K^−^). In addition, we analyzed single-trial brain network dynamics and compared RTs between trials dominated by different brain regions. Further, to search for converging evidence, we sorted trials by RTs within each feedback condition and examined changes in network dynamics as a function of transient changes in performance. The following are the details of materials and methods.

### Ethics Statement

This study was carried out in strict accordance with the recommendations in the Guide for Committee of Laboratory Care and Use of the National Chiao Tung University. The Institutional Review Board of the Taipei Veterans General Hospital approved the protocol. Each participant read and signed an informed consent form before the experiment began.

### Experimental setting

Participants were instructed to maintain an alcohol- and caffeine-restricted diet for one day before each experiment. Each session was performed in the mid-afternoon when subjects were likely to experience somnolence and lasted for approximately 1.5 hr. The monetary compensation for the two sessions was NT$1,200 (approximately US$ 40).

### Virtual-reality (VR) driving environment

Since 2005, we (Brain Research Center, National Chiao Tung University, Taiwan), have conducted a series of fundamental explorations related to simulated driving (such as behavioral lapse^27^,^33^,^34^,^35^,^36^, dual-task^37^,^38^, navigation^39^, motion sickness^40^,^41^, auditory^42^,^43^ and kinesthetic^17^ feedback) and neuroimaging technology^44^,^45^ in a virtual-reality driving environment^46^. The driving simulator allows repeated simulation of emergent situations, such as a sudden stop of the lead vehicle, abrupt lane departure, and simulated crashes, without risking the safety of the driver. Additionally, the repeated-trials approach helps to systematically establish the line between driving performance and changes in EEG activity during continuous driving under a controlled experimental condition.

The VR-based high-fidelity driving environment was established using six identical projectors and PCs that ran the same VR program and were synchronized over local area network. The traffic scenario was projected at viewing angles of 0°, 42°, 84°, 180°, 276° and 318° to provide a 360° visual field. The dimensions of each directional scene were 300 × 225 (width × height) cm, 290 × 225 cm, 260 × 195 cm, 520 × 195 cm, 260 × 195 cm, and 290 × 225 cm, respectively. In this study, the experimental scenario involved a visually monotonous and unexciting nighttime driving on a straight four-lane highway without any other traffic. The refresh rate of the scenario frame was set to emulate cruising at a speed of 100 km/hr. The distance from the left side to the right side of the road was quantized into values of 0–255; the width of each lane was 60 units and that of the car was 28 units, reflecting the ratio of the width of a real lane (3.75 m) to that of a real car (1.8 m). A real vehicle frame (Make: Ford; Model: Probe) that included no unnecessary weight such as an engine, wheels, and other components, was mounted on a six degree-of-freedom Stewart motion platform. In each K^+^ session, the platform actively delivered to the subject the sensation of motion in sync with the driving scene. The platform was inactive during the K^−^ session.

### Experimental paradigm

The event-related lane-departure paradigm, designed by Huang^15^, was implemented in the dynamic driving simulator (see Supplementary Fig. 1). It was designed to mimic a non-ideal road surface that caused the car to drift, with equal probability, to the right or left of the third lane. Following each perturbation (deviation onset), the subject was required to steer the car (response onset) back to the center of the original lane (response offset) as quickly as possible. The sequence between the deviation onset and the response offset constitute one trial. The inter-trial interval (time between response offset and subsequent deviation onset) was selected uniformly at random to be within the range 5–10 s. The driving task continued for 1 hr. with no intervention. The driver’s activities were monitored from the scene control room via a surveillance video camera mounted on the dashboard. Hundreds of lane-departure trials were collected in each of the experiments (see Supplementary Table 1).

### Subjects and sessions

Ten undergraduate and graduate students from National Chiao Tung University, Hsinchu, Taiwan, were recruited to participate in the virtual driving study. All students had valid driving licenses. At the beginning of the experiment, a five-minute pre-test was performed to ensure that every subject understood the instructions and to confirm that they did not suffer from simulator-induced nausea. Each subject was required to complete a questionnaire about his or her sleeping habits. The participants therefore had normal work and rest, normal sleep (around 8hrs of sleep each night), and had not stayed up late (no later than 11:00 pm) at any time in the week before the experiment.

Each subject was asked to participate in one K^−^ session and one K^+^ session on different days. Each session lasted for about 60 min. The order of the K^−^ session and the K^+^ session was counterbalanced across subjects, i.e., half of the participants performed the K^−^ session first followed by the K^+^ session and the other half of participants performed the K^+^ session first followed by the K^−^ session.

### EEG data acquisition

The Synamps2 system (Neuroscan, Inc.) and Ag/AgCl electrodes were used to record 30-channel EEG signals (see Supplementary Fig. 2) with two reference channels placed on the right and left mastoids with a 16-bit quantization level at a sampling rate of 500 Hz. These electrodes were arranged on the quick-cap according to a modified international 10–20 system. The impedance of all of electrodes was kept to less than 5 kΩ.

### Behavioral data collection and task performance measurement

Subjects were instructed to put their best effort to stay alert and respond to the lane-deviation events as soon as possible. The response time (RT), which was the period between the deviation onset and the response onset, was determined as a measure of the subjects’ task performance in the simulated driving task. A highly vigilant subject should respond promptly to lane perturbations (short RTs), while a subject with a low level of vigilance would respond slowly (long RTs). In the absence of any subject response, the vehicle would come into contact with the right or left limits of the roadside within 1.5 and 2.5 s, respectively. The vehicle would then continue to move forward along the roadside until the subject regained consciousness and/or responded to correct the error. The vehicle trajectory and the RT were recorded throughout the experiment (see Supplementary Fig. 3). The mean RTs were calculated for all sessions (see Supplementary Table 1).

### EEG data preprocessing

As shown in Supplementary Fig. 4, the raw EEG data were bandpass-filtered from 1 to 50 Hz using a zero-phase FIR filter (the eegfilt.m routine from the EEGLAB toolbox) to remove high-frequency electrical artifacts (such as line noise) and low-frequency drift, before being downsampled to 250 Hz for data reduction. Data were preprocessed using the EEGLAB toolbox^47^ in MATLAB (Mathworks Inc., Natick, MA).

### EEG source information flow analysis

First, infomax independent component analysis^19^ (EEGLAB’s runica.m function) was performed on EEG recordings to separate instantaneously quasi-independent brain processes from artificial and physiological noise^48^. Then, all quasi-independent brain processes were spatially localized in the brain using a single equivalent current dipole model^49^ based on an assumption that the field dynamics of a small region of homogeneously oriented and spatiotemporally coherent cortical pyramidal cells can be modeled as an equivalent current dipole^49^. For the quasi-independent brain process with a bilaterally symmetric component map (i.e., occipital component map, the rightmost subplot of Supplementary Fig. 5a), a symmetric dual-dipole model was used as suggested by Makeig*, et al.*^50^. ICA has been demonstrated to be effective in identifying sources. The 3D-equivalent brain dipole was obtained by applying the DIPFIT function (using a four-shell spherical model) of the EEGLAB toolbox to the topography of each ICA component (see Supplementary Fig. 5)^47^.

The task-negative or default mode network (DMN) consists of a set of multiple interacting subsystems including the medial prefrontal cortex, medial temporal lobe, posterior cingulate cortex, and retrosplenial cortex^11^,^12^,^51^,^52^,^53^ with the posterior cingulate cortex functioning as a major hub. The DMN is predominantly activated and functionally coherent when one is in a resting state and no explicit task is being performed, when attention is directed toward internal states, thought processes, memories, or feelings, and during performance of a highly over-learned or low-demand task (e.g., driving down a highway under low traffic conditions in a familiar or monotonous environment). The task-positive^12^ or frontal-parietal attention network^13^,^14^ consists of a set of multiple interacting subsystems including a ventral system associated with bottom-up attention processes (temporoparietal cortex and inferior frontal cortex) and a dorsal system associated with top-down attention processes (intraparietal cortex, superior frontal cortex, middle temporal complex, extrastriate cortex) with the middle cingulate cortex functioning as a major hub^54^,^55^. The frontal-parietal attention network is predominantly activated and functionally coherent during performance of tasks requiring attention to sensory-perceptual information from the external environment, especially during performance of a novel or high-demand task (e.g., driving under high traffic conditions in an unfamiliar environment). These two neural systems have been observed to be anti-correlated^12^; however they have also been observed to exhibit flexible coupling between nodes of each system during task performance^56^. Thus, the brain’s large-scale networks may interrelate in a dynamic, flexible and adaptive manner to function either cooperatively or competitively depending on circumstances or context^8^,^9^,^10^.

Independent brain sources obtained from all of the subjects were semi-automatically grouped into distinct clusters with a high intra-cluster similarity^47^. We clustered independent sources using EEGLAB routines implementing k-means clustering on vectors comprised of scalp topographies, equivalent dipole source locations, and time-frequency properties^47^, followed by a manual adjustment to remove outliers. In this study, the processes of independent sources whose spatial coordinates were in or close to the above-mentioned cortical areas were selected as the independent brain sources of interest. For group analysis, six brain sources (see Supplementary Fig. 5) consistently observed across 20 experiment sessions (10 subjects × 2 conditions) were selected for further analysis. These sources were labeled as anterior cingulate cortex (ACC), midcingulate cortex (MCC), left sensorimotor cortex (lSMC), right sensorimotor cortex (rSMC), posterior cingulate cortex (PCC), and extrastriate cortex (ESC) according to the mean locations in Talairach space.

Granger causality refers to the fact that signal *X*_1_ can cause another signal *X*_2_ if the information in the past of *X*_1_ helps predict the future of *X*_2_. We can represent the multivariate process at time *t* as a stationary autoregressive process of order *p* as follows. Consider, for example, *n* = 3.


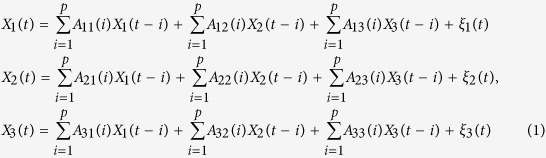


where 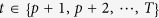 is the current time point and *T* is the length (in samples) of the signal. The model order *p* was estimated by applying the Bayesian information criterion (BIC). Parameters *A* and 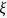 are respectively the model coefficient matrix and prediction error, which are obtained using ordinary least squares approach^57^.

In this study, the multivariate linear dynamical (autoregressive) model was fit to the process activation time series in a 1-s window before the lane departure (i.e., pre-perturbation period). Before the model fitting, detrending and centering (subtraction of the temporal mean) were performed to remove linear drift present in the data.

Following the model fitting, the model is validated by 1) the whiteness of the residuals, 2) the percentage of the consistency, and 3) the stationarity of the model. The validation results of all experimental sessions are shown in Supplementary Table 2. Then, the fitted model was used to infer effective connectivity by means of time-domain conditional multivariate Granger causality analysis^20^,^58^.

To determine the causal effect of *X*_2_ on *X*_1_, the prediction error was re-estimated using a submodel (restricted) that excluded the signal *X*_2_; the value thus obtained was compared with that obtained using the full (unrestricted) model.


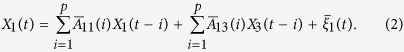


The strength of the effect of *X*_2_ to *X*_1_ (i.e., causal magnitude), conditioned on *X*_3_, was determined as follows.


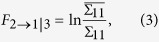


where 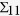 and 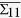 are the variance of 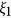 and 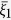, respectively.

In this study, the Granger causality of *X*_2_ on *X*_1_ is tested in the context of multiple additional variables 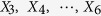.

### Statistical analyses

Finally, statistical significance with respect to a null hypothesis of zero connectivity was assessed for each pair of regions using a nonparametric surrogate statistical method^58^ by the following procedure.

1. Construct a distribution, 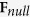, satisfying the null distribution of no connectivity using a phase-randomization approach. The sample size of surrogate data is 2000.
2. Compare the observed connectivity magnitude, 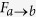, with the quantiles of the null distribution to obtain the *p*-value:
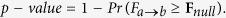
3. Adjust 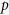-values using the false discovery rate (FDR)-controlling multiple testing procedure^59^ (the fdr.m routine from the EEGLAB toolbox^47^). The FDR-adjusted 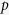-values < 0.01 are marked in grey (see Supplementary Table 3), indicating the connectivity magnitude is significantly different from zero.
4. Calculate the number of significant outgoing edges (i.e., out-degree) between brain regions.
5. Obtain the proportion of subjects for the edge 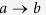
which has a significantly non-zero information flow, P(edge)(see Supplementary Table 3).
6. High agreement across participants in terms of significantly non-zero connectivity (i.e., P(edge) ≥ 0.9) was visualized in an anatomically-coregistered 3D space.
7. Perform McNemar’s test followed by the FDR correction to determine whether kinesthetic feedback has an effect on the connectivity (i.e., the proportion of individuals whose connectivity differed when driving with kinesthetic feedback as opposed to driving without kinesthetic feedback). The results of all edges were shown in 2 × 2 contingency tables (see Supplementary Table 4).

MATLAB toolboxes, including EEGLAB^47^, the source information flow toolbox (SIFT, an EEGLAB-compatible toolbox)^60^, and Granger causal connectivity analysis (GCCA toolbox)^61^, were used to analyze and visualize multivariate causality and the flow of information between sources of electrophysiological activity.

### Network-sorted RT comparison

Single-trial Granger causality analysis was also performed to characterize the network dynamics (measured by out-degree) directly correlated with behavioral variability during tasks. All trials were sorted by the causal hub, i.e., the region with the highest out-degree. The trials were discarded in case of a tie. Then, the percentage of these classified trials was calculated followed by a two-way repeated measures ANOVA (2 × 2 design) to analyze the influence of sensory feedback and brain region factors on the percentages. The RT of trials dominated by the different causal hub was compared as shown in Supplementary Table 5.

### RT-sorted out-degree dynamics

The performance-sorted out-degree was also introduced to observe the continuous changes of out-degree from fast to slow RT. For each trial, all the out-degrees were normalized by dividing the sum of significant outflow links of all brain regions. Then, the normalized out-degrees are horizontally stacked according to the RT from fast to slow and smoothed with a central moving average filter (window size: 10% of trials; window step: 1 trial).

### RT-sorted spectral dynamics

Each of the 1-s (250-points) pre-stimulus MCC and PCC activations was transformed into a time-frequency representation by applying the fast Fourier transform (FFT) with Welch’s method and Hanning window. All of the 250-point data were first divided into overlapping 128-point segments, windowed with Hanning window. Then, all segments were zero-padded to 256 points for a 256-point FFT, resulting in an estimate of the power spectral density with 30 frequency bins from 0.98 to 30.3 Hz. The log power spectra were sorted in order of ascending RT and then concatenated into a 2D image (x-axis: frequency and y-axis: RT). The mean log power spectrum (in dB) of the trials with an RT below the tenth percentile of RTs, was used as the reference value and subtracted from each estimated spectrum.

## Additional Information

**How to cite this article**: Lin, C.-T. *et al.* Mind-Wandering Tends to Occur under Low Perceptual Demands during Driving. *Sci. Rep.* **6**, 21353; doi: 10.1038/srep21353 (2016).

## Supplementary Material

Supplementary Information

## Figures and Tables

**Figure 1:**
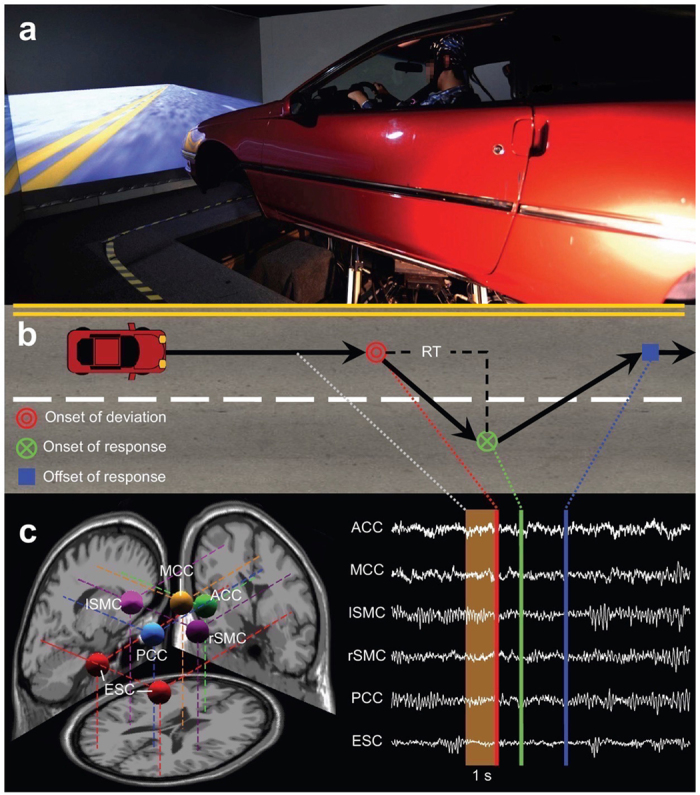
Environmental setting and EEG sources. (**a**) A participant driving the simulated vehicle while EEG is recorded. A six-degree-of-freedom Stewart platform underneath the floor supports the simulator and modulates the orientation of the vehicle to provide the sensation of external motion. (**b**) Simultaneous behavioral and EEG data were collected throughout the experiment, in which the event-related lane-departure driving paradigm was implemented. Each trial involved three essential events − onset of deviation, onset of response, and offset of response − which were recorded in chronological order. (**c**) The selected six regions of interest (left panel) and sample time courses of activation (right panel). Effective connectivity measures were derived from independent EEG activations 1-s before onset of perturbations to compute causal relationship between brain regions.
