# Supplementary material for: Mind-Wandering Tends to Occur under Low Perceptual Demands during Driving
Source: Sci Rep. 2016 Feb 17;6:21353. doi: 10.1038/srep21353 (PMC4808905; doi:10.1038/srep21353)
Supplement: Supplementary Information [file srep21353-s1.pdf]

Supplementary Information

Mind-Wandering Tends to Occur under Low Perceptual Demands during Driving

Chin-Teng Lin*, Chun-Hsiang Chuang*, Scott Kerick, Tim Mullen,
Tzyy-Ping Jung, Li-Wei Ko, Shi-An Chen, Jung-Tai King, Kaleb McDowell

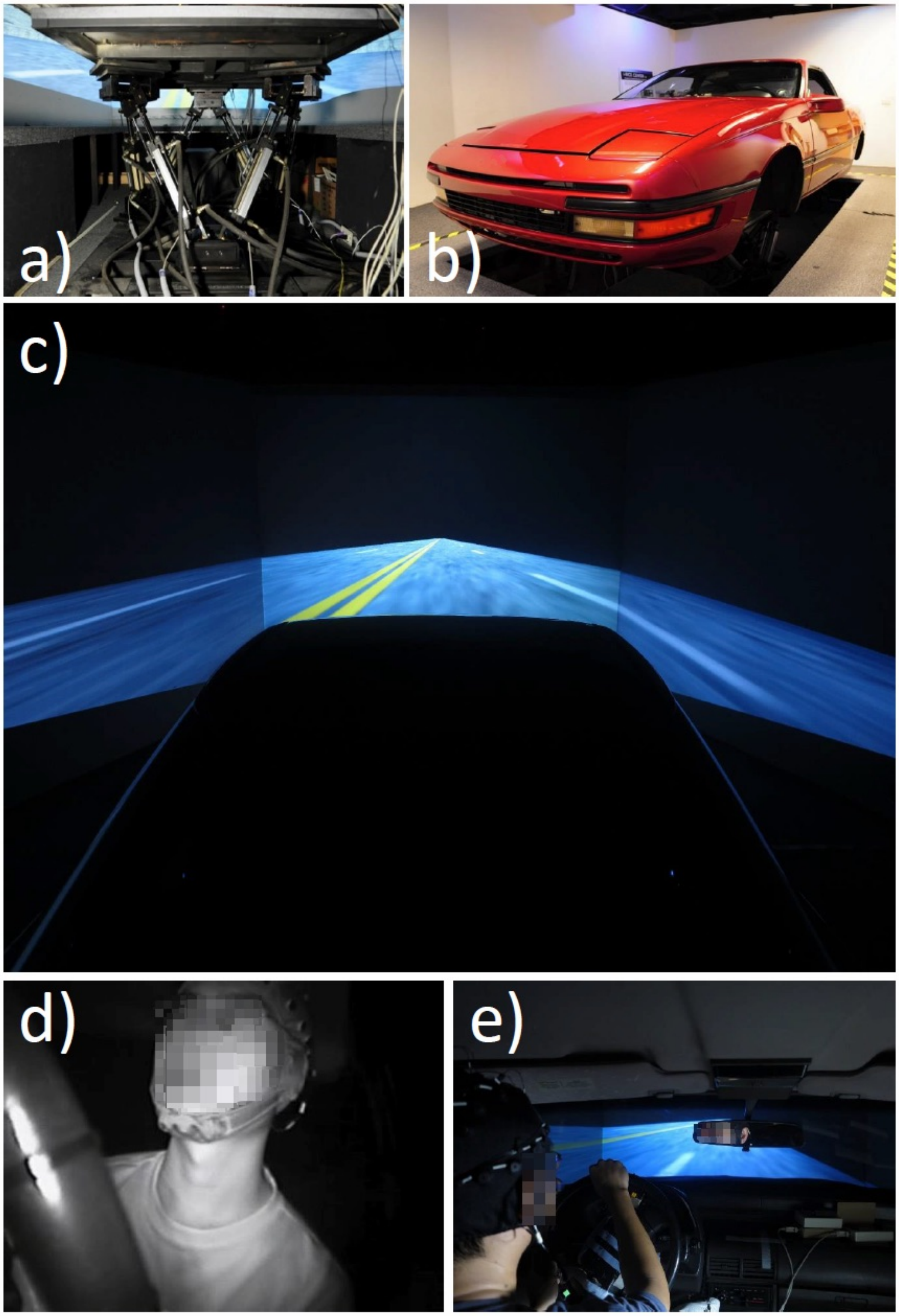

<u>Supplementary Figure 1.</u> Virtual-reality (VR) driving environment. a, Six degree-of-freedom Stewart motion platform. b, Real vehicle frame mounted on the platform. c, Simulated nighttime driving. d, A snapshot of the surveillance video. e, Drivers view of oncoming highway from inside cabin.

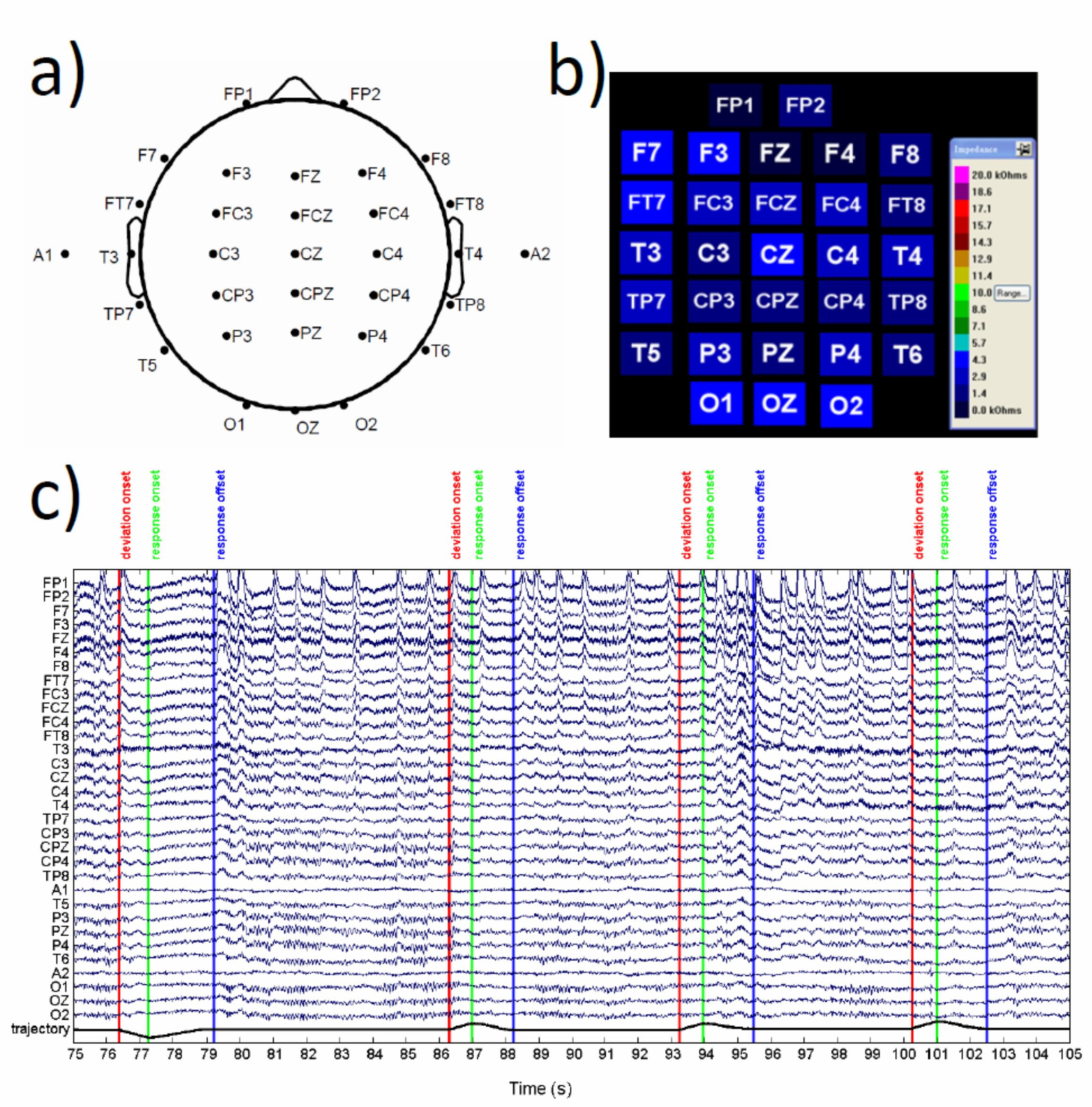

Supplementary Figure 2. **EEG setting. a,** Placement of 30-channel EEG electrodes on the scalp. Channels A1 and A2 are reference channels. **b,** Impedance of all of electrodes. **c,** Simultaneous EEG (first 30 channels) and vehicle trajectory (last channel) recordings. Red, green, and blue lines represent the events of deviation onset, response onset, and response offset, respectively.

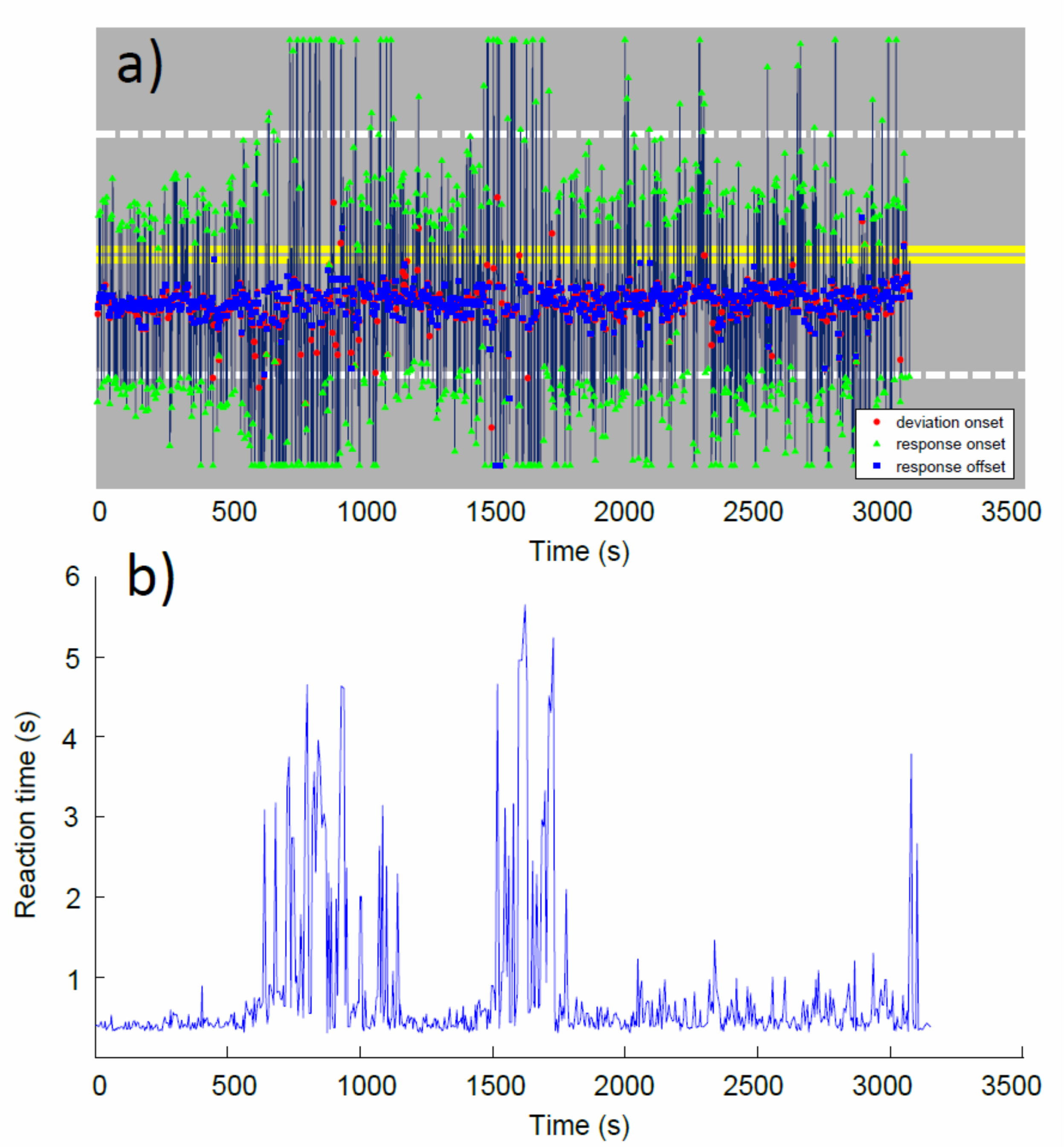

Supplementary Figure 3. Behavioral data of a single participant. a, Vehicle trajectory and b, reaction time.

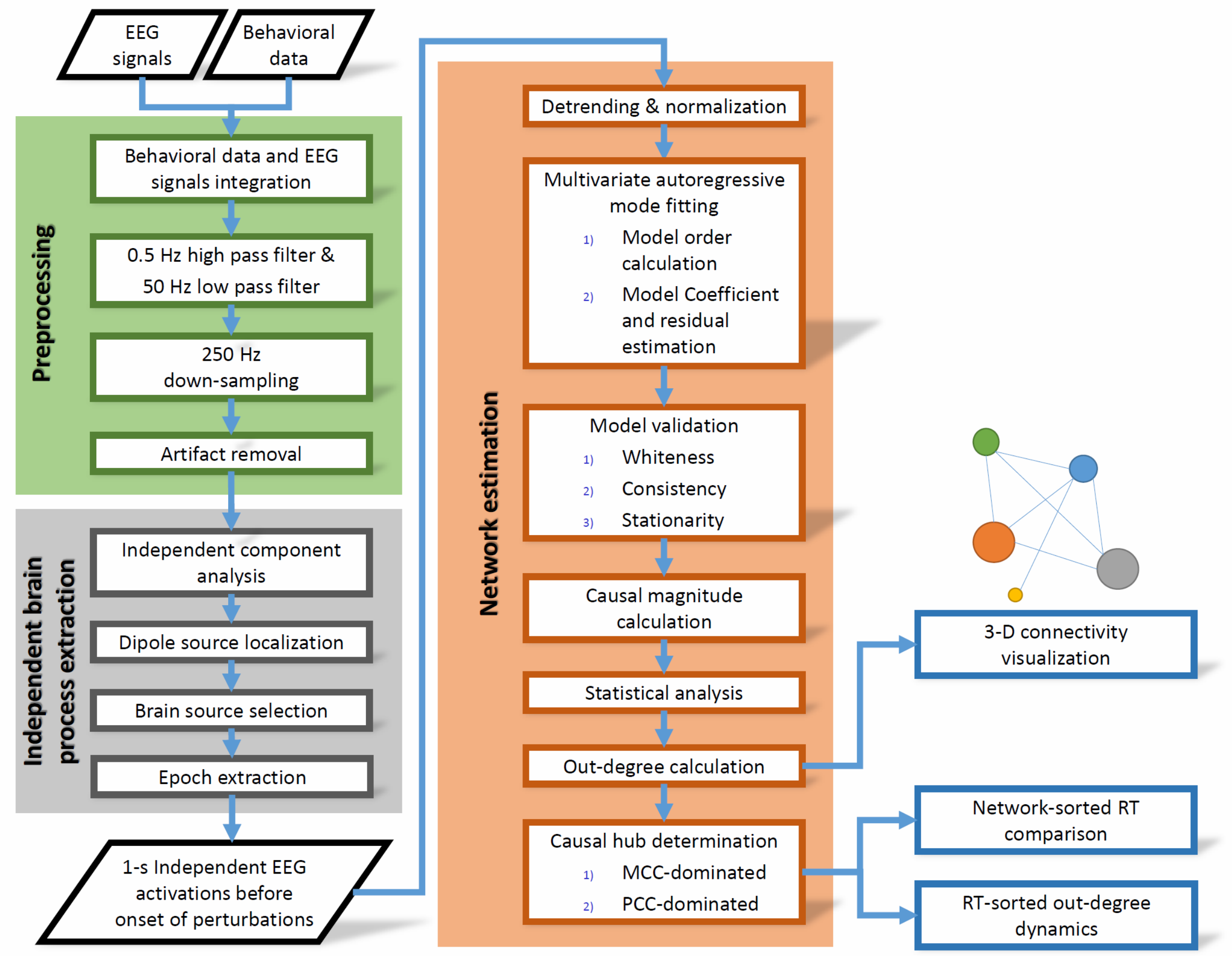

Supplementary Figure 4. Flowchart of effective connectivity estimation.

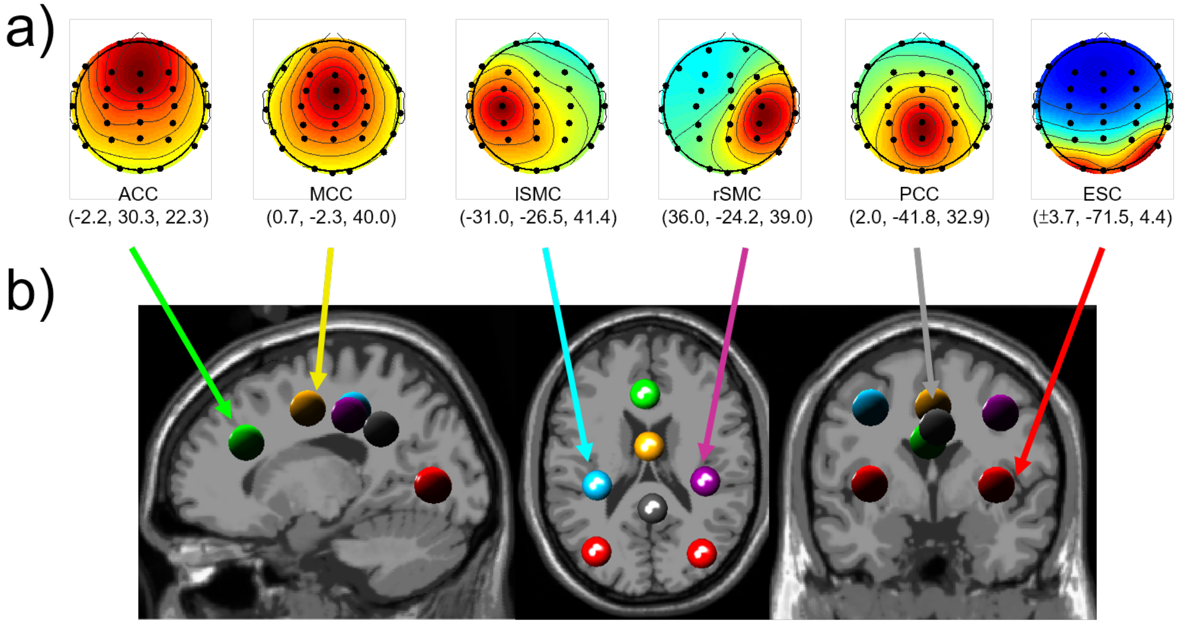

<u>Supplementary Figure 5.</u> Independent brain sources of interest. a, Mean 2-D topographic maps and Talairach coordinates of six independent sources. These maps reveal the strength of independent processes in the active brain, projected onto the scalp. b, 3-D equivalent dipole source localizations of six independent components; images (left to right) correspond to viewing along sagittal, horizontal, and coronal planes. These nodes are approximate sources of each independent component, and the large marker indicates the mean location. Nodes are color-coded by component. Green: anterior cingulate cortex (ACC); yellow: midcingulate cortex (MCC); blue: left sensorimotor cortex (lSMC); purple: right sensorimotor cortex (rSMC); white: posterior cingulate cortex (PCC); red: extrastriate cortex (ESC).

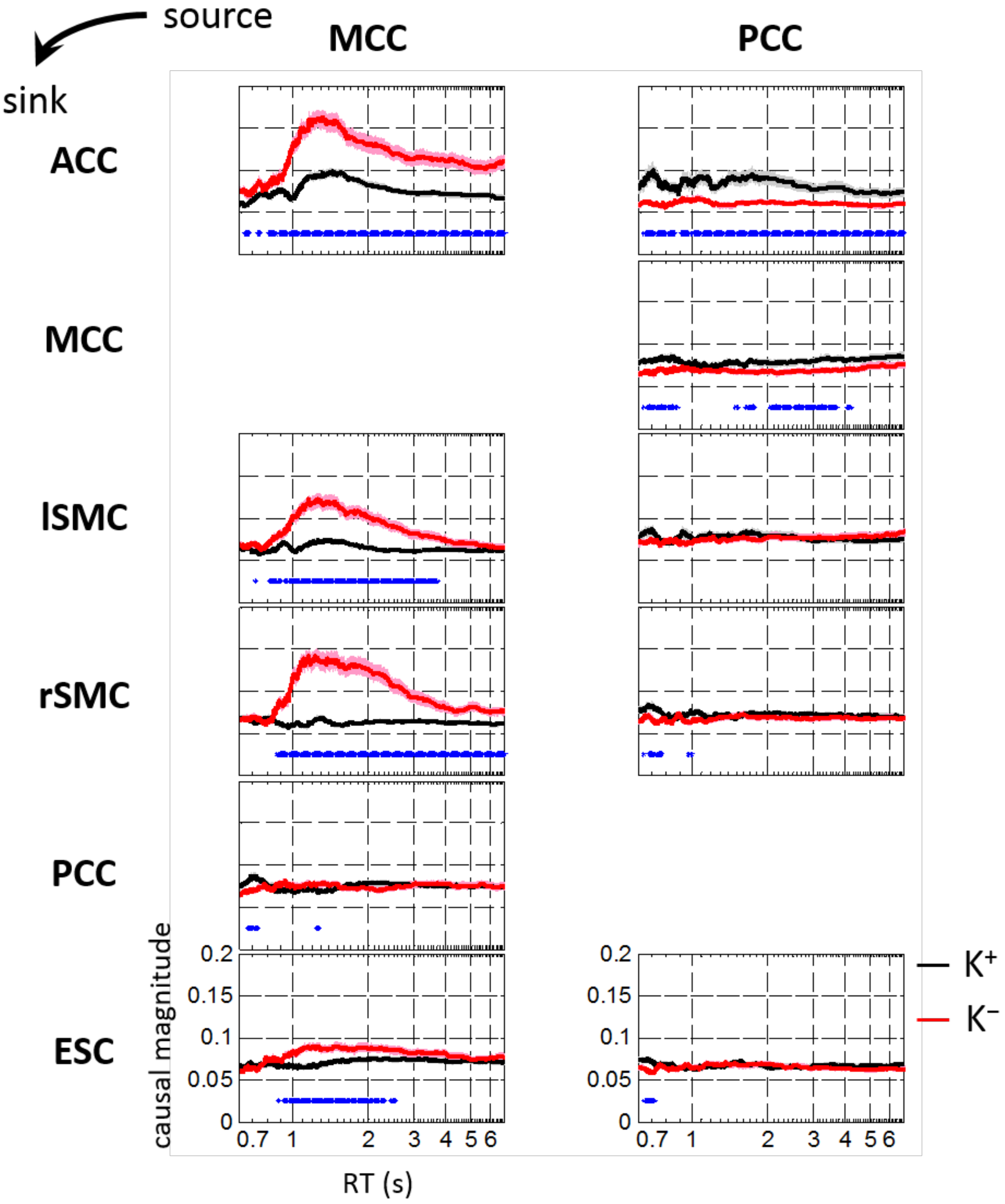

Supplementary Figure 6. Connectivity between pairs of brain regions, where causality is from columns to rows. Each cell plots changes in connectivity magnitude from fast to slow RT under K^+ (black trace) and K^- (red trace) conditions. Blue asterisks indicate the statistically significant difference of the causal magnitude between two conditions. An inverted-U shaped change of the connectivity magnitude occurred in MCC→ACC, MCC→left SMC and MCC→right SMC, but only under the K^- condition. This result might indicate that strengthening the connectivity from MCC to motor areas is necessary to compensate for the declining performance when the motion feedback is not available.

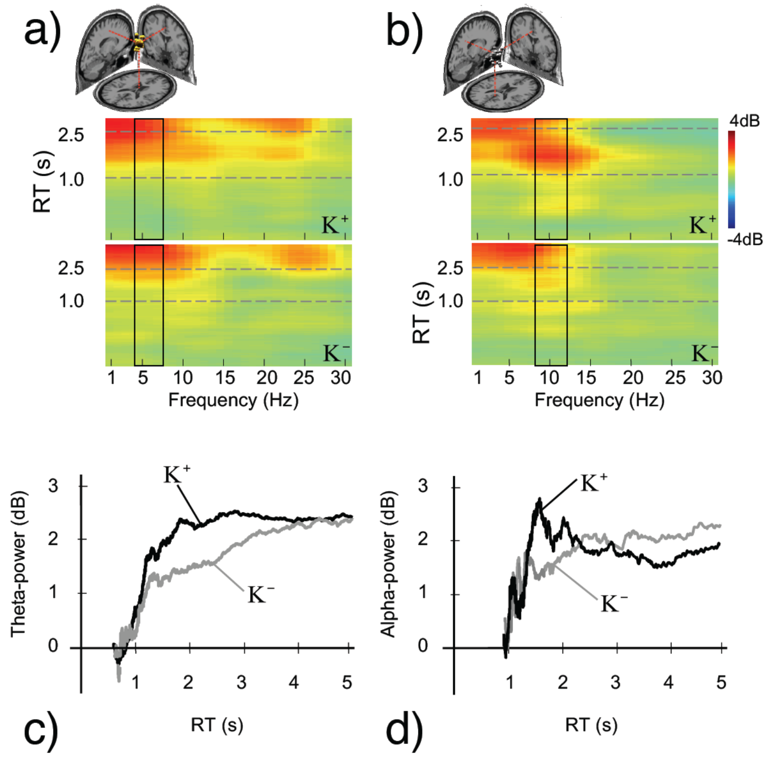

<u>Supplementary Figure 7.</u> Power spectra co-varied with RT. Power perturbations of a, MCC and b, PCC regions; upper and lower panels concern K^+ and K^- conditions, respectively. Each colored horizontal bar represents a single trial in a spectral estimate (1-30 Hz). c, RT-sorted mean theta power (4-7 Hz) of MCC, and d, RT-sorted mean alpha power (8-12 Hz) of PCC under K^+ (black trace) and K^- (grey trace) conditions. As RT increased, the EEG power in the 1-5 Hz frequency monotonically increased in MCC and PCC under both K^+ (the upper panel) and K^- (the lower panel) conditions. The K^+ condition was associated with biphasic neural activity from 5-15 Hz, in which the spectral power was greatest when task performance was suboptimal (1-2.5 s). Supplementary Figure 7c compares the average theta power (4-7 Hz) in MCC between the K^+ (black trace) and K^- (gray trace) conditions. In the RT range of 1-4 s, MCC had higher theta power under the K^+ condition than under the K^- condition. Supplementary Figure 7d compares the average alpha-power (8-12 Hz) in PCC between the two conditions. Compared to the K^- condition, a higher alpha power was observed under the K^+ condition when the RT was in the range of 1.3-2.2 s, and a less alpha power was observed under the K^+ condition when the RT exceeded 2.2 s.

Supplementary Table 1. List of reaction time under K^+ and K^- conditions.

		Number of trials		Reaction time (s)	
		K^+	K^-	K^+	K^-
Subject	**S01**	315	404	1.613 ± 2.014	1.334 ± 1.287
	**S02**	465	319	2.233 ± 2.460	2.756 ± 3.043
	**S03**	430	259	1.380 ± 0.676	1.590 ± 1.158
	**S04**	242	159	1.501 ± 2.342	1.460 ± 2.799
	**S05**	277	312	1.735 ± 2.161	3.045 ± 3.845
	**S06**	424	275	2.325 ± 2.528	2.745 ± 4.039
	**S07**	245	241	0.964 ± 1.660	1.406 ± 2.324
	**S08**	198	191	1.371 ± 1.964	1.377 ± 1.910
	**S09**	229	226	1.143 ± 1.006	1.172 ± 1.198
	**S10**	162	94	0.763 ± 0.305	1.695 ± 3.466
**Total/Mean ± standard deviation**		**2987**	**2480**	**1.503 ± 0.503**	**1.858 ± 0.702**

<u>Supplementary Table 2.</u> MVAR model order selection and validation tests.

		K^+				K^-			
		**Model order**	**Model validation test**			**Model order**	**Model validation test**		
			**Whiteness[1,2]**	**Consistency[3]**	**Stationarity[4]**		**Whiteness[1,2]**	**Consistency[3]**	**Stationarity[4]**
**Subject**	**S01**	6-7	100%	88.0±6.5%	100%	6-7	100%	89.8±6.1%	100%
	**S02**	6-7	100%	87.9±4.9%	100%	6	100%	87.3±5.3%	100%
	**S03**	6-7	100%	87.8±4.7%	100%	5-6	100%	84.6±5.3%	100%
	**S04**	5-6	100%	80.7±8.8%	100%	5-6	100%	83.7±5.4%	100%
	**S05**	6-7	100%	87.4±5.5%	100%	6-7	100%	89.6±4.3%	100%
	**S06**	6-8	100%	92.0±5.3%	100%	6-7	100%	91.5±4.9%	100%
	**S07**	5-6	100%	86.7±5.6%	100%	5-6	100%	87.1±4.9%	100%
	**S08**	5-6	100%	81.8±8.3%	100%	5-6	100%	81.7±7.4%	100%
	**S09**	6-7	100%	87.2±6.2%	100%	5-6	100%	83.8±7.8%	100%
	**S10**	6-7	100%	90.7±5.6%	100%	5-6	100%	86.4±6.6%	100%

1. The percentage shown in the whiteness and stationarity tests represents the proportion of trials that meet the requirement of the test.
2. The Durbin-Watson test was used for checking the whiteness of model residuals at a Bonferroni-corrected significant level of $p < 0.05/6 = 0.0083$.
3. The consistency shows the average portion (± standard deviation) of the correlation structure in data is accounted for by an MVAR model.
4. The augmented Dickey-Fuller test was used for model stationarity test.

<u>Supplementary Table 3.</u> Individual and cross-subject results of the effective connectivity.

K^+		**FDR-adjusted *p*-values[1]**										**P(edge)[2]**	**Out-degree[3]**
**Source→Sink**		**S01**	**S02**	**S03**	**S04**	**S05**	**S06**	**S07**	**S08**	**S09**	**S10**		
	**MCC**	0.0098	0.0098	0.0138	0.0000	0.0098	0.0055	0.0077	0.0098	0.0098	0.0077	**0.9**	
	**lSMC**	0.0098	0.0098	0.0098	0.0077	0.0156	0.0098	0.0098	0.0098	0.0098	0.0098	**0.9**	
**ACC**	**rSMC**	0.0110	0.0110	0.0123	0.0175	0.0098	0.0098	0.0098	0.0123	0.0098	0.0098	0.5	3
	**PCC**	0.0098	0.0098	0.0098	0.0098	0.0890	0.0098	0.0098	0.0098	0.0098	0.0098	**0.9**	
	**ESC**	0.0123	0.0110	0.0123	0.0110	0.0123	0.0123	0.0123	0.0098	0.0123	0.0110	0.1	
	**ACC**	0.0098	0.0098	0.0123	0.0000	0.0098	0.0055	0.0077	0.0098	0.0000	0.0055	**0.9**	
	**lSMC**	0.0123	0.0123	0.0123	0.0110	0.0110	0.0123	0.0110	0.0138	0.0098	0.0123	0.1	
**MCC**	**rSMC**	0.0110	0.0138	0.0123	0.0123	0.0110	0.0098	0.0098	0.0098	0.0098	0.0110	0.4	3
	**PCC**	0.0098	0.0098	0.0098	0.0098	0.0098	0.0077	0.0098	0.0098	0.0098	0.0098	**1.0**	
	**ESC**	0.0098	0.0098	0.0098	0.0098	0.0098	0.0098	0.0098	0.0098	0.0055	0.0098	**1.0**	
	**ACC**	0.0196	0.0175	0.0156	0.0138	0.0175	0.0123	0.0098	0.0156	0.0175	0.0138	0.1	
	**MCC**	0.0123	0.0123	0.0123	0.0138	0.0098	0.0110	0.0098	0.0110	0.0110	0.0123	0.2	
**lSMC**	**rSMC**	0.0123	0.0123	0.0123	0.0156	0.0098	0.0123	0.0110	0.0123	0.0098	0.0110	0.2	0
	**PCC**	0.0110	0.0098	0.0098	0.0110	0.0123	0.0110	0.0098	0.0098	0.0098	0.0123	0.5	
	**ESC**	0.0098	0.0110	0.0098	0.0098	0.0123	0.0110	0.0110	0.0110	0.0098	0.0098	0.5	
	**ACC**	0.0123	0.0098	0.0156	0.0110	0.0123	0.0098	0.0098	0.0098	0.0110	0.0098	0.5	
	**MCC**	0.0123	0.0196	0.0138	0.0156	0.0123	0.0123	0.0123	0.0123	0.0156	0.0221	0.0	
**rSMC**	**lSMC**	0.0123	0.0110	0.0123	0.0110	0.0098	0.0123	0.0123	0.0138	0.0123	0.0123	0.1	0
	**PCC**	0.0098	0.0098	0.0098	0.0098	0.0098	0.0098	0.0098	0.0110	0.0098	0.0248	0.8	
	**ESC**	0.0098	0.0098	0.0098	0.0098	0.0098	0.0098	0.0110	0.0123	0.0110	0.0138	0.6	
	**ACC**	0.0138	0.0098	0.0110	0.0098	1.0000	0.0098	0.0098	0.0098	0.0098	0.0098	0.7	
	**MCC**	0.0175	0.0098	0.0098	0.0055	0.0098	0.0077	0.0098	0.0098	0.0098	0.0098	**0.9**	
**PCC**	**lSMC**	0.0098	0.0098	0.0098	0.0098	0.0221	0.0098	0.0055	0.0098	0.0098	0.0098	**0.9**	4
	**rSMC**	0.0098	0.0098	0.0098	0.0098	0.0098	0.0098	0.0098	0.0098	0.0098	0.0138	**0.9**	
	**ESC**	0.0098	0.0098	0.0098	0.0098	0.0098	0.0098	0.0110	0.0098	0.0098	0.0098	**0.9**	
	**ACC**	0.0156	0.0221	0.0175	0.0175	0.0138	0.0175	0.0156	0.0175	0.0196	0.0248	0.0	
	**MCC**	0.0110	0.0098	0.0098	0.0098	0.0098	0.0098	0.0098	0.0098	0.0098	0.0098	**0.9**	
**ESC**	**lSMC**	0.0098	0.0098	0.0098	0.0098	0.0098	0.0098	0.0098	0.0098	0.0098	0.0123	**0.9**	2
	**rSMC**	0.0123	0.0098	0.0110	0.0123	0.0098	0.0123	0.0123	0.0156	0.0098	0.0110	0.3	
	**PCC**	0.0123	0.0196	0.0156	0.0156	0.0110	0.0156	0.0175	0.0138	0.0123	0.0196	0.0	

1. The shaded cells indicate significantly non-zero information flows (FDR-adjusted $p < 0.01$).
2. P(edge) denotes the proportion of subjects which have significant connectivity amplitude for that edge. The number in bold denotes P(edge) ≥ 0.9.
3. The out-degree indicates the number of outgoing edges.

Supplementary Table 3. Individual and cross-subject results of the effective connectivity (continued).

K^{-}		FDR-adjusted *p*-values[1]										P(edge)[2]	Out-degree[3]
**Source→Sink**		**S01**	**S02**	**S03**	**S04**	**S05**	**S06**	**S07**	**S08**	**S09**	**S10**		
**ACC**	**MCC**	0.0000	0.0000	0.0000	0.0000	1.0000	0.0000	0.0000	0.0000	0.0000	0.0000	**0.9**	3
	**lSMC**	0.0055	0.0000	0.0110	0.0000	0.0098	0.0055	0.0077	0.0098	0.0077	0.0000	**0.9**	
	**rSMC**	0.0098	0.0098	0.0559	0.0110	0.0196	0.0098	0.0098	0.0077	0.0098	0.0098	0.7	
	**PCC**	0.0055	0.0098	0.0098	0.0098	1.0000	0.0098	0.0098	0.0098	0.0055	0.0098	**0.9**	
	**ESC**	0.0098	0.0055	0.0175	0.0098	1.0000	0.0110	0.0098	0.0098	0.0110	0.0098	0.6	
**MCC**	**ACC**	0.0098	0.0098	0.0098	0.0098	1.0000	0.0098	0.0077	0.0098	0.0055	0.0055	**0.9**	5
	**lSMC**	0.0000	0.0000	0.0123	0.0000	0.0000	0.0000	0.0000	0.0000	0.0000	0.0000	**0.9**	
	**rSMC**	0.0000	0.0000	0.0351	0.0000	0.0000	0.0000	0.0000	0.0000	0.0000	0.0000	**0.9**	
	**PCC**	0.0098	0.0098	0.0098	0.0098	0.0443	0.0098	0.0098	0.0098	0.0098	0.0098	**0.9**	
	**ESC**	0.0055	0.0000	0.0098	0.0077	0.0175	0.0077	0.0077	0.0000	0.0055	0.0000	**0.9**	
**lSMC**	**ACC**	0.0196	0.0110	0.0175	0.0110	0.0196	0.0110	0.0098	0.0123	0.0123	0.0138	0.1	2
	**MCC**	0.0055	0.0098	0.0221	0.0055	0.0098	0.0098	0.0098	0.0077	0.0098	0.0077	**0.9**	
	**rSMC**	0.0123	0.0098	0.0175	0.0098	0.0110	0.0123	0.0110	0.0098	0.0098	0.0110	0.4	
	**PCC**	0.0098	0.0055	0.0098	0.0098	0.0098	0.0098	0.0110	0.0098	0.0098	0.0098	**0.9**	
	**ESC**	0.0175	0.0123	0.0138	0.0138	0.0110	0.0175	0.0138	0.0175	0.0156	0.0123	0.0	
**rSMC**	**ACC**	0.0123	0.0123	0.0628	0.0138	0.0175	0.0098	0.0123	0.0138	0.0138	0.0138	0.1	1
	**MCC**	0.0077	0.0055	0.0351	0.0055	0.0055	0.0000	0.0000	0.0000	0.0098	0.0077	**0.9**	
	**lSMC**	0.0123	0.0098	0.0175	0.0123	0.0098	0.0110	0.0098	0.0098	0.0098	0.0098	0.6	
	**PCC**	0.0110	0.0098	0.0110	0.0098	0.0098	0.0098	0.0110	0.0098	0.0098	0.0110	0.6	
	**ESC**	0.0138	0.0098	0.0156	0.0098	0.0098	0.0123	0.0098	0.0110	0.0098	0.0098	0.6	
**PCC**	**ACC**	0.0156	0.0156	0.0138	0.0138	0.0628	0.0138	0.0138	0.0138	0.0156	0.0110	0.0	0
	**MCC**	0.0123	0.0098	0.0098	0.0098	0.0351	0.0138	0.0098	0.0098	0.0098	0.0123	0.6	
	**lSMC**	0.0123	0.0098	0.0098	0.0077	0.0110	0.0110	0.0098	0.0098	0.0098	0.0098	0.7	
	**rSMC**	0.0175	0.0110	0.0123	0.0156	0.0110	0.0110	0.0123	0.0123	0.0123	0.0138	0.0	
	**ESC**	0.0175	0.0098	0.0098	0.0098	0.0110	0.0098	0.0098	0.0098	0.0098	0.0156	0.7	
**ESC**	**ACC**	0.0175	0.0098	0.0098	0.0098	0.0110	0.0098	0.0098	0.0098	0.0098	0.0156	0.7	1
	**MCC**	0.0098	0.0098	0.0098	0.0098	0.0196	0.0098	0.0098	0.0098	0.0098	0.0098	**0.9**	
	**lSMC**	0.0123	0.0123	0.0123	0.0123	0.0098	0.0123	0.0123	0.0123	0.0123	0.0123	0.1	
	**rSMC**	0.0098	0.0098	0.0123	0.0098	0.0123	0.0110	0.0123	0.0098	0.0098	0.0098	0.6	
	**PCC**	0.0110	0.0110	0.0110	0.0098	0.0110	0.0110	0.0098	0.0098	0.0098	0.0098	0.5	

1. The shaded cells indicate significantly non-zero information flows (FDR-adjusted $p < 0.01$).
2. P(edge) denotes the proportion of subjects which have significant connectivity amplitude for that edge. The number in bold denotes P(edge) ≥ 0.9.
3. The out-degree indicates the number of outgoing edges.

<u>Supplementary Table 4.</u> 2x2 Contingency Tables of all edges.

ACC→MCC (FDR-*p*=1.000)

		K^-	
		y	n
K^+	y	8	1
	n	1	0

ACC→lSMC (FDR-*p*=1.000)

		K^-	
		y	n
K^+	y	8	1
	n	1	0

ACC→rSMC (FDR-*p*=1.000)

		K^-	
		y	n
K^+	y	4	1
	n	3	2

ACC→PCC (FDR-*p*=1.000)

		K^-	
		y	n
K^+	y	9	0
	n	0	1

ACC→ESC (FDR-*p*=0.071)

		K^-	
		y	n
K^+	y	1	0
	n	5	4

MCC→ACC (FDR-*p*=1.000)

		K^-	
		y	n
K^+	y	8	1
	n	1	0

MCC→lSMC (FDR-*p*=0.035*)

		K^-	
		y	n
K^+	y	1	0
	n	8	1

MCC→rSMC (FDR-*p*=0.071)

		K^-	
		y	n
K^+	y	4	0
	n	5	1

MCC→PCC (FDR-*p*=1.000)

		K^-	
		y	n
K^+	y	9	1
	n	0	0

MCC→ESC (FDR-*p*=1.000)

		K^-	
		y	n
K^+	y	9	1
	n	0	0

lSMC→ACC (FDR-*p*=1.000)

		K^-	
		y	n
K^+	y	1	0
	n	0	9

lSMC→MCC (FDR-*p*=0.035*)

		K^-	
		y	n
K^+	y	2	0
	n	7	1

lSMC→rSMC (FDR-*p*=1.000)

		K^-	
		y	n
K^+	y	1	1
	n	3	5

lSMC→PCC (FDR-*p*=1.000)

		K^-	
		y	n
K^+	y	4	1
	n	5	0

lSMC→ESC (FDR-*p*=0.071)

		K^-	
		y	n
K^+	y	0	5
	n	0	5

rSMC→ACC (FDR-*p*=1.000)

		K^-	
		y	n
K^+	y	1	4
	n	0	5

rSMC→MCC (FDR-*p*=0.035*)

		K^-	
		y	n
K^+	y	0	0
	n	9	1

rSMC→lSMC (FDR-*p*=0.071)

		K^-	
		y	n
K^+	y	1	0
	n	5	4

rSMC→PCC (FDR-*p*=1.000)

		K^-	
		y	n
K^+	y	5	3
	n	1	1

rSMC→ESC (FDR-*p*=1.000)

		K^-	
		y	n
K^+	y	3	3
	n	3	1

PCC→ACC (FDR-*p*=0.035*)

		K^-	
		y	n
K^+	y	0	7
	n	0	3

PCC→MCC (FDR-*p*=1.000)

		K^-	
		y	n
K^+	y	6	3
	n	0	1

PCCC→lSMC (FDR-*p*=1.000)

		K^-	
		y	n
K^+	y	7	2
	n	0	1

PCC→rSMC (FDR-*p*=0.035*)

		K^-	
		y	n
K^+	y	0	9
	n	0	1

PCC→ESC (FDR-*p*=1.000)

		K^-	
		y	n
K^+	y	6	3
	n	1	0

ESC→ACC (FDR-*p*=0.035*)

		K^-	
		y	n
K^+	y	0	9
	n	0	1

ESC→MCC (FDR-*p*=1.000)

		K^-	
		y	n
K^+	y	8	1
	n	1	0

ESC→lSMC (FDR-*p*=0.035*)

		K^-	
		y	n
K^+	y	1	8
	n	0	1

ESC→rSMC (FDR-*p*=1.000)

		K^-	
		y	n
K^+	y	2	1
	n	4	3

ESC→PCC (FDR-*p*=0.071)

		K^-	
		y	n
K^+	y	0	0
	n	5	5

1. The sign of y and n denote the non-zero and zero connectivity, respectively.
2. The numbers display the total number of individuals whose connectivity is **a.** (upper-left cell) non-zero in both K^+ and K^- conditions, **b.** (upper-right cell) non-zero in the K^+ condition and zero in the K^- condition, **c.** (lower-left cell) zero in the K^+ condition and non-zero in the K^- condition, **d.** (lower-right cell) zero in both K^+ and K^- conditions.

<u>Supplementary Table 5.</u> Reaction time of MCC- and PCC-driven network.

Reaction time (s)		K^+		K^-	
		MCC-driven	PCC-driven	MCC-driven	PCC-driven
**subject**	**S01**	1.616	1.687	1.278	1.404
	**S02**	2.210	2.405	2.689	2.821
	**S03**	1.348	1.477	1.495	1.975
	**S04**	1.628	1.183	0.919	1.870
	**S05**	1.455	1.762	2.902	3.093
	**S06**	2.365	2.446	2.136	2.892
	**S07**	1.129	0.833	1.745	1.265
	**S08**	1.165	1.512	1.049	1.832
	**S09**	1.013	1.328	1.163	1.077
	**S10**	0.787	0.706	0.946	1.687
